# Complex Pathophysiological Mechanisms and the Propose of the Three-Dimensional Schedule For Future COVID-19 Treatment

**DOI:** 10.3389/fimmu.2021.716940

**Published:** 2021-10-20

**Authors:** Yonggang Zhou, Xiuxiu Xu, Haiming Wei

**Affiliations:** ^1^ Institute of Gerontology, The First Affiliated Hospital of University of Science and Technology of China (USTC), Division of Life Sciences and Medicine, University of Science and Technology of China, Hefei, China; ^2^ Hefei National Laboratory for Physical Sciences at Microscale, The Chinese Academy of Sciences (CAS) Key Laboratory of Innate Immunity and Chronic Disease, Division of Life Sciences and Medicine, University of Science and Technology of China, Hefei, China; ^3^ Institute of Immunology, University of Science and Technology of China, Hefei, China

**Keywords:** COVID-19, ARDS, Inflammatory storm program, Inflammatory cytokine, Inflammatory cell death

## Abstract

At present, the global COVID-19 epidemic is still in a state of anxiety, and increasing the cure rate of critically ill patients is an important means to defeat the virus. From an immune perspective, ARDS driven by an inflammatory storm is still the direct cause of death in severe COVID-19 patients. Although some experience has been gained in the treatment of COVID-19, and intensive COVID-19 vaccination has been carried out recently, it is still effective to save lives to develop more effective programs to alleviate the inflammatory storm and ARDS in patients with SARS-CoV-2 or emerging variants of SARS-CoV-2. In reorganizing the ARDS-related inflammatory storm formation program in COVID-19 patients, we highlighted the importance of the vicious circle of inflammatory cytokines and inflammatory cell death, which is aggravated by blood circulation to form multi-system inflammation. Summarizes the interlocking and crisscrossing of inflammatory response and inflammatory cell death mechanisms including NETs, pyrolysis, apoptosis and PANoptosis in severe COVID-19. More importantly, in response to the inflammatory storm formation program we described, and on the premise of following ethical and clinical experimental norms, we propose a three-dimensional integrated program for future research based on boosting antiviral immune response at the initial stage, inhibiting inflammatory cytokine signaling at the exacerbation stage and inhibiting cell death before it’s worse to prevent and alleviate ARDS.

## 1 Introduction

In the past 20 years, various coronaviruses including severe acute respiratory syndrome coronavirus (SARS-CoV) in 2003 (1), middle east respiratory syndrome coronavirus (MERS-CoV) in 2012 (2) and SARS-CoV-2 in 2020 (3, 4) have caused many health crises in different countries and regions around the world. The genome sequence of SARS-CoV-2 has nearly 80% homology with SARS-CoV and about 50% with MERS-CoV (5). In the latest and more extensive screening, samples of pneumonia patients collected from Sarawak Regional Hospital in Malaysia in the past few years were found to be canine-derived coronavirus infections (6) and in Haitian children, pig-derived delta coronaviruses were also found (7). The global pandemic of acute infectious pneumonia named “Coronavirus disease 2019 (COVID-19)”caused by SARS-CoV-2, is by far the most widespread, longest lasting and worst example among them.

From the beginning of 2020 to the present, the COVID-19 epidemic has risen and fallen throughout the world in just over a year, and the latest round of the epidemic caused by the rapid and continuous mutation of SARS-CoV-2 has also broken out (8). Globally, as of 16 September 2021, there have been exceeded 226 million confirmed cases of COVID-19, including >4.65 million deaths, reported to the World Health Organization (WHO). As a result, the overall prevalence of mortality in COVID-19 patients was ~2% (4.65/226). Although billions doses of vaccination that have been completed worldwide have brought hope to control the epidemic, the speed of vaccination is still a few days away from the universal immune barrier. The recent report of the centers for disease control and prevention in the United States also showed cases of COVID-19 vaccine breakthrough infections. And recent studies have also shown that existing vaccines, including mRNA vaccines, adenovirus vaccines, inactivated vaccines and RBD-subunit vaccines, have reduced the neutralizing activity against the SARS-CoV-2 mutant strains (9, 10). In addition, we still lack specific anti-SARS-CoV-2 drugs. Therefore, while developing a broader-spectrum vaccine and wonder drugs, we should further study the mechanism of death caused by COVID-19 and develop more effective treatment options.

The spectrum of COVID-19 presentations ranges from the asymptomatic infection, to a mild self-limiting influenza-like illness, to life-threatening multiorgan failure (11–13). Most COVID-19 patients present mild or moderate symptoms, about 15% of patients develop severe pneumonia and 5% progress to critically ill (14–16). So, reducing the incidence of multiorgan failure is the key to improve the cure rate and reduce the mortality of COVID-19 (17, 18). A large number of inflammatory macrophage infiltration and the distribution of inflammatory cytokines such as interleukin (IL)-1β, IL-6, and IL-18, were found in the pulmonary pathology of patients with severe COVID-19 (19–21). The inflammatory storm instigated by pathogenic T cells and inflammatory monocytes was considered to be the key to the severity of COVID-19 (22). These cells release proinflammatory factors represented by granulocyte-macrophage colony-stimulating factor (GM-CSF) and IL-6, which recruit more inflammatory cells into the lungs and other organs to form a “cytokine release syndrome”, and the further aggravated inflammatory storm will eventually lead to multiorgan failure and death in patients with severe COVID-19 (22–24). Based on these basic findings, the COVID-19 immunotherapy strategy that targets these inflammatory cytokines or their receptors to relieve the inflammatory storm have benefited patients with COVID-19 in the past year. The results of a Chinese study exploring the treatment of tocilizumab for COVID-19 including 21 patients in the intensive care unit (ICU) were the first to encourage this treatment strategy (25). Clinical trials focusing on blocking IL-6 signaling to treat COVID-19 benefit patients, including IL-6 receptor antagonists [tocilizumab (26, 27) and sarilumab (28)] and IL-6 inhibitors [siltuximab (29)], and tocilizumab needs to be combined with standard antiviral care to be highlighted in the comparison of international multi-center clinical trials (26, 30). Subsequent clinical trials of monoclonal antibody drugs showed that treatments targeting the GM-CSF receptor (mavrilimumab) and the IL-1 receptor (anakinra) were also related to clinical improvement of patients with severe COVID-19 (31, 32). Although these monoclonal antibody drugs that target inflammatory cytokine signals have shown some benefits, they were limited by many complex factors such as drug targets, the treatment time, the dosage, and differences in patient immune responses, and their performance in reducing patient mortality was unsatisfactory (24, 33, 34).

To further improve the COVID-19 immunotherapy strategy to better reduce the risk of patient death, it is necessary to re-analyze the process of COVID-19 inflammatory storm based on recent research findings. Here, we discuss the progression of acute respiratory distress syndrome (ARDS), a typical evolution of severe COVID-19, as a starting point, reorganize the process of severe inflammatory storms, and try to propose a targeted graded treatment plan for a future research based on the combination of antiviral immune response, inflammatory immune response and inflammatory cell death. Although targeting each individual aspect of this three-dimensional program has been shown to be effective, it has to be said that the overall treatment plan is still an idealized strategy. Therefore, to test its superiority and earlier application, we call for the three-dimensional graded treatment plan to be considered for clinical trials under the premise of ethical requirements.

## 2 ARDS Is a Life-Threatening Condition of COVID-19 Induced by Inflammatory Storm

ARDS is a common cause of respiratory failure in critically ill patients and is a severe pulmonary condition that leads to refractory hypoxemia (35, 36). Alveolar surfactant is a foamy substance that can keep the full expansion of the alveoli, which is essential for breathing. In ARDS, lung injury causes fluid to leak into the space between the alveoli and capillaries, and as the pressure increases, fluid builds up inside the alveoli to accumulate and degrade surfactants, forming a typical ARDS characteristic—accumulation of fluid in the lungs, causing the alveoli to collapse (36). These changes prevent the lungs from filling properly with air, disrupting the gas exchange in the lungs, and causing a series of serious cascade reactions that impact the oxygen supply of tissues and organs. Because of this, ARDS usually occurs in life-threatening conditions such as severe pneumonia, sepsis and severe trauma. The incidence of ICU patients worldwide is about 10%, and the mortality rate is as high as 30-40% (37, 38).

Viral infections, especially coronavirus and avian influenza virus (H5N1), cause pneumonia to be one of the main factors leading to ARDS (39). In the coronavirus epidemic caused by the SARS-CoV for the first time in 2003, reports showed that the incidence of ARDS was about 25% (40). ARDS had also occurred in some severe cases and animal models with MERS infection (41). A study from the early days of the COVID-19 epidemic showed that nearly 40% of severe and critical hospitalized patients developed ARDS, and more than half of those diagnosed died from the disease (42). So, ARDS is closely related to death caused by coronavirus infection. In those patients with ARDS who recovered, although the lung function gradually improved within a year or so, it was difficult to recover as before, lung volume was below normal, and scarring was present (14, 42).

In patients with symptomatic coronavirus infection, pulmonary inflammation was activated, and pneumonia develops into ARDS as the inflammation worsens (22, 40–42). More and more evidences suggest that the occurrence of ARDS seems to be less directly due to the infected virus itself and more related to excessive rather than effective inflammation in the body (15, 43, 44). This excessive inflammation is usually manifested as the continuous release of inflammatory factors, which is aggravated by blood circulation to form multi-system inflammation, which is called cytokine storm or inflammatory storm (22–24, 45).

## 3 Inflammatory Storm Program That Triggers ARDS in COVID-19

**Figure 1:**
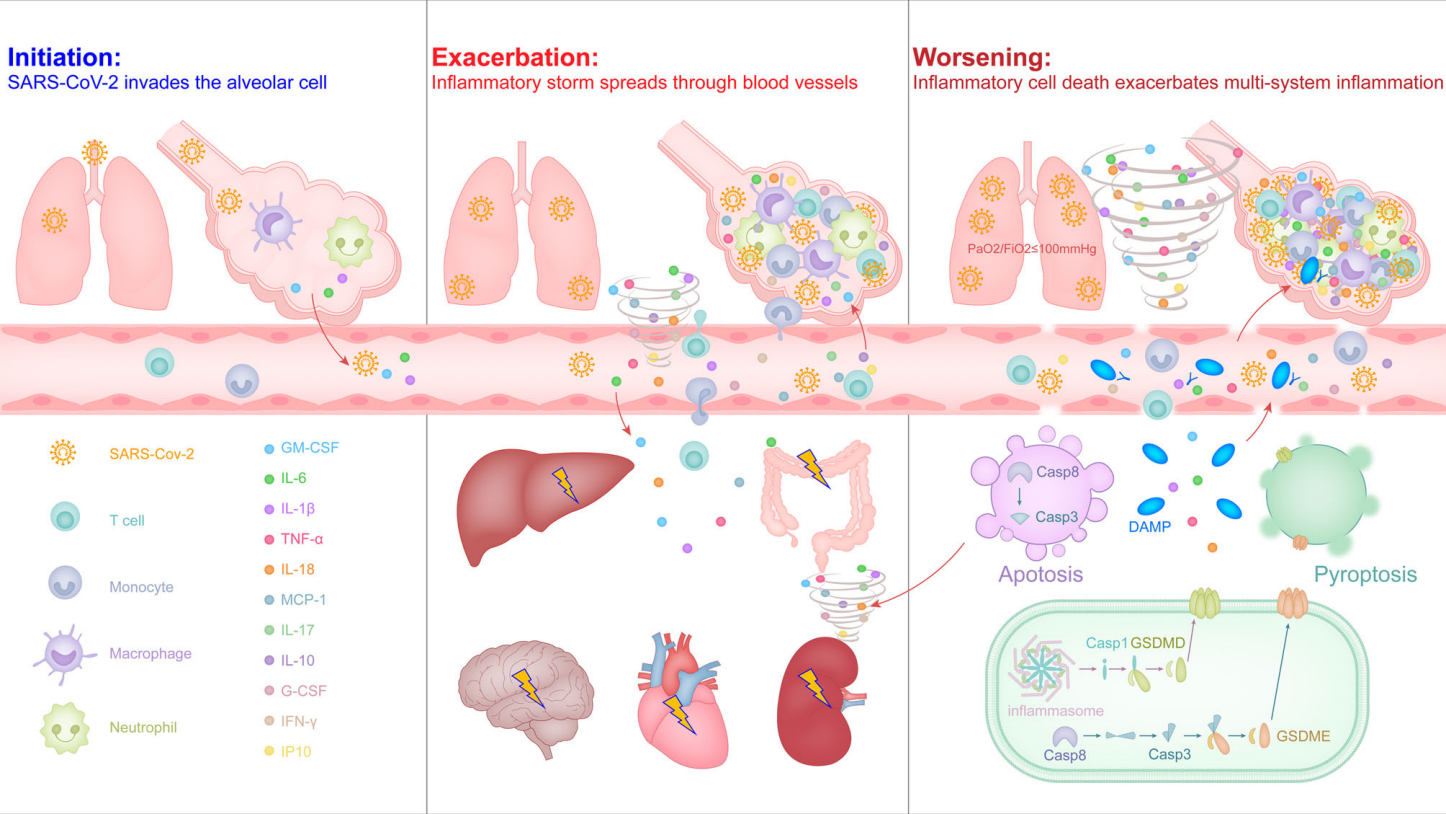
The malignant cycle of inflammatory factors and inflammatory cell death exacerbates the inflammatory storm to trigger ARDS and multiorgan failure in severe COVID-19.

Similar to the common influenza virus, the SARS-CoV-2 enters the respiratory tract of most people, it will also activate the antiviral immune response that causes inflammation, leading to mild symptoms such as sore throat, cough, fever. In some cases, the virus is difficult to control and escapes into the alveoli to stimulate excessive release of inflammatory factors, triggering an inflammatory storm and developing ARDS (**Figure 1**).

### 3.1 Initiation: SARS-CoV-2 Invades the Type II Alveolar Epithelial Cell

Respiratory droplets are the main carrier of the SARS-CoV-2, and its journey begins in the nose, mouth and eyes, and travels down the alveoli in the lungs (46, 47). SARS-CoV-2 is an enveloped, positive-sense single-stranded RNA virus, which belongs to *Betacoronavirus* genus and is highly pathogenic (48, 49). SARS-CoV-2 encodes four structural proteins, among which the nucleocapsid (N) protein combines with RNA to form a helical capsid, spike (S), envelope (E), and membrane (M) constitute the viral membrane proteins, of which spike mediates virus entry into host cells (49–51). In the study of SARS-CoV, it has been confirmed that the main receptor of S protein is angiotensin-converting enzyme 2 (ACE2) expressed in type II alveolar epithelial cells (52). The S protein coding gene of SARS-CoV-2 is highly variable with SARS-CoV, and the nucleotide homology is less than 75% (5, 51). The S protein is trimeric-like clover-shaped, with three S1 heads and one trimeric S2 stem, and the receptor-binding domain (RBD) is located at the tip of each S1 head (49, 52, 53). After the RBD in the S protein mediates direct contact with ACE2 on the target cell surface, the transmembrane serine protease (TMPRSS2) cleaves the C-terminal peptide of ACE2 to enhance the virus invasion driven by the S protein (54). In addition, recent studies have shown that the CD147 molecule that can be expressed on most leukocytes, platelets and endothelial cells is also the host receptor for the RBD in S protein of SARS-CoV-2, participating in the interaction between the virus and the target cell and helping the virus invade (55). When the virus successfully infects a type II alveolar epithelial cell, it will inject its own RNA into the cell and achieve a large amount of replication, releasing more virus to infect other target cells nearby.

Due to the need to defend against pathogenic microorganisms brought in by breathing air, the liquid layer on the alveolar surface resides with immune cells, especially macrophages with phagocytic function, which account for more than 95% and are called alveolar macrophages (56). SARS-CoV-2 may directly infect these myeloid cells by binding to the C-type lectin receptor on the surface of cells *via* the glycosylation sites in the non-RBD region of the S protein, and this recognition mode did not induce the antiviral immune response of interferon, but instead led to the release of a large number of inflammatory factors (57). Macrophages can be polarized into pro-inflammatory M1 macrophages or M2 macrophages that inhibit inflammation, depending on the stimulus conditions they receive. Under physiological conditions, alveolar macrophages exhibit an anti-inflammatory M2 phenotype (56, 58). Recent studies have shown that the endosomal vesicles of M2 type macrophages are slightly alkaline, which can inhibit the separation of SARS-CoV-2 nucleic acid from viral particle components and help lysosomes to degrade the virus (59). This may be one of the reasons why most infected people have mild symptoms and can effectively control the virus in the early stage. In some severe cases, the out-of-control virus induces alveolar cells to release cytokines and higher proportions of M1 macrophages and neutrophils in the bronchoalveolar lavage fluid (60), intended to activate a stronger antiviral immune response, but it also produces a strong inflammatory response and alveolar injury (**Figure 1**, left). M1 type macrophages are softer and have better phagocytic effects, but the endosomal vesicles of M1 macrophages is acidic, which helps the SARS-CoV-2 nucleic acid to be separated from the viral particle components, thereby helping the virus amplification (20, 59). In addition, it also increases the risk of the virus spreading from M1 macrophages into the blood throughout the body.

### 3.2 Exacerbation: Inflammatory Storm Spreads Through Blood Vessels

The increased inflammation in COVID-19 patients leads to a further increase in body temperature and inflammation-related clinical indicators, such as C-reactive protein, serum ferritin, and IL-6 (13, 16). As inflammation and viruses spread to the blood, T cells are rapidly activated, and over-activated T cells develop into pathogenic T cells, producing factors such as GM-CSF and IL-6 (22). GM-CSF further activates CD14^+^CD16^+^ inflammatory monocytes to produce a larger amount of IL-6 and other inflammatory factors (e.g., IL-1β, IL-8, IL-18, and TNF-α), thereby forming an inflammatory storm, leading to severe immune damage in the lungs and other organs (15, 22, 23). Most patients with severe COVID-19 are diagnosed with lymphopenia based on blood routine reports, especially T cells (13, 16). This is not only related to apoptosis or death caused by syncytia after excessive activation of T cells (61), but may also be related to inflammatory infiltration of lungs and other organs. In the histopathological examination of the lungs, heart, intestines, etc. of critically ill patients, significant inflammatory cell infiltration was observed, including inflammatory macrophages, neutrophils, and pathological T cells (19, 20, 62).

Contrary to lymphopenia, the increase of neutrophils in the capillaries or inflammatory tissues of infected patients is also a sign of the severity of COVID-19 (13, 14), and most inflammatory factors can promote the activation of neutrophils (63). Activated neutrophils release cytokines and chemokines, and the networked DNA-protein complex structure forms neutrophil extracellular traps (NETs) to trap and kill pathogenic microorganisms (63, 64). During the formation of NETs, a variety of intracellular damage-associated molecular patterns (DAMPs) are released, activating pattern recognition receptors, causing the surrounding immune or non-immune cells to produce excessive pro-inflammatory cytokines and chemokines; and those released together include histone, DNA, myeloperoxidase (MPO), neutrophil elastase, cathepsin and proteinase-3 and other granular proteins, which cause increasing tissue necrosis (64). In severe COVID-19 patients, NETs-related signaling pathways in lung tissue are up-regulated, and the level of MPO-DNA complex in plasma is higher, suggesting lung tissue damage and platelet-triggered NETs formation is related (65, 66). Activated neutrophils can also activate complement by releasing NETs to cause endothelial damage and necrotizing inflammation, and further promote venous thrombosis (64). In addition, NETs can activate platelets through extracellular DNA and provide a scaffold for the combination of red blood cells and activated platelets, thereby promoting a wider connective network and amplifying the formation of immune thrombi (65, 67). Indeed, a recent study showed that SARS-CoV-2 can also directly infect vascular endothelial cells with the accumulation of inflammatory monocytes (e.g., neutrophils) in multiple organs of patients with severe COVID-19, such as lungs, heart, kidney, small intestine and liver (68). Patients with severe COVID-19 also have clinical symptoms of disseminated intravascular coagulation with elevated serum D-dimer and prolonged prothrombin times (14, 16, 69). Together, it is reasonable to believe that the direct attack of the virus and the infiltration of inflammatory immune cells caused by the infiltration of vascular endothelial cells will loosen the tight junctions of vascular endothelial cells, thereby promoting the spread of vascular leakage and inflammatory storms to multiple organs throughout the body through the circulatory system, and further aggravating lung damage (**Figure 1**, middle).

### 3.3 Worsening: Inflammatory Cell Death Exacerbates Multi-System Inflammation

Although cell death (e.g., pyroptosis, apoptosis, and necroptosis) is an important mechanism for controlling pathogenic microbial infections, inflammatory cell death also leads to the release of inflammatory factors and cell contents, including alarmins and DAMPs, which causes severe inflammatory responses (64, 70).

Pyroptosis is a form of inflammatory cell death that is mediated by the caspases-inflammasome or -gasdermin cascade, which manifests as the continuous expansion of cells until the cell membrane ruptures, resulting in the release of cell contents and activating a strong inflammatory response (71). Pyrolysis is also the main mechanism for the release of non-signal peptide inflammatory factors, such as the release of IL-1β or IL-18 depends on the caspase-1-dependent gasdermin D cascade (72). In the lung pathology and peripheral blood from patients with severe COVID-19, it was also observed that the pyrolysis of macrophages led to the release of the IL-1β and IL-18 by NLRP3 inflammasome activation and cleavage of caspase-1 (20, 73, 74).

Apoptosis was originally thought to be a non-inflammatory form of cell death, which breaks down cells through membrane vesicles to avoid direct release of cell contents. However, more and more recent evidence shows that due to the crosstalk between the caspase family of apoptotic proteins and the gasdermin family of lysing cell executors, apoptosis is not always inflammatory silent (75–77). For example, in the caspase cascade that drives the onset of apoptosis, caspase 3 can cleave gasdermin E and caspase 8 can cleave gasdermin D to lyse cells under special conditions (75, 77), such as the ORF3a protein stimulation of SARS-CoV-2 (78). SARS-CoV-2 can also induce airway epithelial cells to show apoptosis and cytopathic characteristics (79).

Compared with the release of NETs by neutrophils, more DAMPs are released due to the inflammatory death of cells induced by thrombus and tissue damage (80). High levels of endogenous DAMP molecule S100A8/A9, HMGB1 and lactate dehydrogenase can be detected in the serum of severe COVID-19 patients (81, 82). The latest reports show that patients with severe COVID-19 produce a large number of autoantibodies against autoantigens including intracellular molecules, which indirectly supports the theory that inflammatory cell death promotes the formation of a hyperinflammatory state (83).

In a study on the effects of inflammatory factors released by COVID-19 on cell death, it was confirmed that tumor necrosis factor α (TNF-α) and interferon γ (IFN-γ), two inflammatory factors that were significantly elevated at the end of the inflammatory response, can induce PANoptosis, a regulated and extensive inflammatory cell death mode, and provide a molecular scaffold for the interaction and activation of mechanisms necessary for pyrolysis, apoptosis and necrosis (76, 84). Together, although more research is needed to fully clarify the inflammatory cell death pathway in the process of SARS-CoV-2 infection and the functional consequences of these processes, more and more evidence is pointing towards this. Due to the spread of the blood circulatory system, a large number of inflammatory factors, DAMPs and alarmins produced by inflammatory cell death completely amplify the inflammatory storm from the lungs into the multi-system of body, which not only makes the lungs worse, but also induces multiple organ failure and causes death that is difficult to save (**Figure 1**, right).

## 4 Proposing the Targeted and Graded COVID-19 Treatment Schedule for a Future Research

### 4.1 Current Progress in COVID-19 Treatment

COVID-19 is a new infectious disease caused by the spread of SARS-CoV-2 mediated through respiratory particles, with complex clinical manifestations, ranging from no symptoms to critical illness associated with respiratory failure, septic shock, and multiorgan failure (14, 85). In the face of an increasing number of severe cases caused by the global spread of SARS-CoV-2, there is an urgent need for experimental therapies and drug repurposing to alleviate the COVID-19. Since the COVID-19 pandemic, global research institutes and hospitals have carried out intensive research work and clinical trials, and developed new treatment methods and multiple vaccines targeting SARS-CoV-2 at an unprecedented speed, making the management of COVID-19 significant progress. Therefore, in addition to symptomatic treatment, there are currently some treatments of proven benefit in antiviral and anti-inflammatory aspects, which are recommended for use under the emergency use authorization (EUA) or further evaluated in licensed clinical trials (86–88).

#### 4.1.1 Small-Molecule Antiviral Agents

Antiviral medications are regarded as the essential requirement to control the outbreak of COVID-19, just like oseltamivir plays an important role in fighting the influenza virus (89). Multiple antiviral agents with anti-SARS-CoV-2 activity identified by *in vitro* screening during the early onset of the pandemic, including remdesivir, hydroxychloroquine and lopinavir/ritonavir, but subsequent randomized controlled clinical trials have shown little or no benefit (90–92). Ivermectin, as a cheap drug approved for antiparasitic use, has recently been reported to have a strong ability to inhibit SARS-CoV-2 replication *in vitro* (93), but unfortunately, the published clinical trial results do not support the conclusion of *in vitro* testing (94). In the latest living guideline of COVID-19 treatments by WHO issued on July 6, 2021, it is clearly recommended to against remdesivir for hospitalized patients with COVID-19 and against hydroxychloroquine, lopinavir/ritonavir or ivermectin for patients with COVID-19 of any severity (88).

#### 4.1.2 Anti-SARS-CoV-2 Neutralizing Antibody Cocktails

Compared with the above-mentioned dilemma of small-molecule antiviral agents, the anti-SARS-CoV-2 neutralizing antibody cocktails have appeared promising in current clinical trials. Neutralizing antibodies, as an important antiviral weapon produced by the immune system, remain in the plasma of individuals recovering from the viral infection. As a traditional antiviral immunotherapy, convalescent plasma therapy was evaluated by clinical trials in China during the early onset of the pandemic (95), and subsequently authorized to be used for critically ill patients with COVID-19 under EUA in the United States (96, 97). This is only a stopgap measure due to the uncertain effects of the other composition from the plasma on therapeutic efficacy and safety. At present, the neutralizing antibody targeting SARS-CoV-2 obtained through recombinant expression technology has entered the stage of clinical trials. REGN-COV2, consisting of two monoclonal antibodies casirivimab and imdevimab, a neutralizing antibody cocktail drug to target the SARS-CoV-2 RBD domain, has been proven to reduce the viral load in the body compared with placebo (98), and it can effectively reduce hospitalization or mortality when administered to non-hospitalized patients with COVID-19 based on public clinical trial data (99). Bamlanivimab/Etesevimab, consisting of a cocktail of neutralizing antibodies targeting Skipe protein of the SARS-CoV-2, also benefits non-hospital patients in clinical trials, reducing hospitalization and mortality (100). Based on these clinical trials, REGN-COV2 and Bamlanivimab/Etesevimab have been licensed to treat non-hospitalized patients with COVID-19 under the EUA in the United States, but as the SARS-CoV-2 variants continue to update, their effectiveness needs further evaluation.

#### 4.1.3 Type I Interferon

Interferon is a cytokine with antiviral and immunomodulatory activities produced by host cells when a virus infects the body, and is seen as the body’s first line of antiviral defence (101). Population studies have found that the COVID-19 severity is related to patients carrying autosomal genetic locus mutations associated with type I IFN genes (102) or the presence of neutralizing autoantibodies against type I IFN in patients (103). Moreover, the lack of type I IFN in the blood may be a potential predictor of the COVID-19 severity (104). These studies have highlighted the important role of type I IFN in the control of SARS-CoV-2 infection, so it is speculated that at least in the early stages of SARS-CoV-2 infection, the use of type I IFN may have therapeutic benefits for some patients. Some preliminary clinical trial data show that compared with the placebo group, inhaled interferon-α or interferon-β can achieve greater clinical improvement, reduce hospital stay and increase the chance of recovery (105, 106).

#### 4.1.4 Antagonists of Inflammatory Factors

Contrary to the low antiviral immune response caused by the lack of type I interferon, the excessive immune response triggers a surge of inflammatory cytokines and the formation of an inflammatory storm that leads to a sudden turn of the disease. IL-1β is the pro-inflammatory cytokine produced by immune cells after recognizing viruses to activate inflammasomes, and it is also increased in COVID-19 patients (20). Anakinra, as an IL-1 receptor antagonist, is a drug approved for the treatment of rheumatoid arthritis and has the potential to reduce the need for invasive mechanical ventilation and mortality in severe COVID-19 patients based on a small case-control study (31). Pathological T cells that produce GM-CSF have been identified in COVID-19 patients (22), and the monoclonal antibody mavrilimumab that blocks the GM-CSF receptor has also shown promising prospects in preliminary clinical trials (32). But overall, the clinical research data targeting the early pro-inflammatory factor IL-1β or GM-CSF is still insufficient, and the efficacy of alleviating the inflammatory storm of COVID-19 still needs further confirmation.

In contrast, IL-6, as the core pro-inflammatory cytokine, has received extensive attention in the research on the inflammatory storm of COVID-19 (22, 33, 45). Three IL-6 signaling antagonists are used to try to alleviate the inflammatory storm of COVID-19, including the monoclonal antibody (Tocilizumab, Sarilumab) that blocks IL-6 receptors authorized for various rheumatological conditions and the monoclonal antibody (Siltuximab) that targets IL-6 authorized for Castleman’s syndrome. Tocilizumab’s confidence in alleviating the inflammatory storm of COVID-19 first began with the preliminary results of an clinical trial of Tocilizumab combined with conventional antiviral drugs in 21 patients with severe COVID-19 (25). Subsequently, the results of a large international multi-center randomized controlled trial (EMPACTA, NCT04372186) showed that for hospitalized patients with COVID-19 who were not mechanically ventilated, adding tocilizumab on the basis of standard care can reduce the risk of mechanical ventilation or death in patients (26). The results of another large randomized controlled trial (REMAP-CAP, NCT02735707) are consistent, and it also showed that treatment with tocilizumab or sarilumab to critically ill COVID-19 patients in the ICU can improve the outcomes including survival (107). Antagonists of IL-6 receptors (Tocilizumab, Sarilumab) have been authorized by the governments of China, the United Kingdom, and the United States to treat COVID-19 patients under EUA. In the latest living guideline of COVID-19 treatments issued by the WHO, tocilizumab or sarilumab is strongly recommended for use in severe and critical COVID-19 (88).

### 4.2 “Combined Boxing” Is Worthy of Consideration in Future COVID-19 Treatment

As mentioned above, we have accumulated some experience in the therapeuqcs of COVID-19, but there are still hundreds of thousands of new confirmed cases of COVID-19 and nearly 10,000 deaths every day in the world according to the data released by WHO. Stress from the frequent occurrence of SARS-CoV-2 variants is a well-known cause, and on the other hand, we also need to face up to the fact that we still haven’t found specific antiviral medicines, especially for SARS-CoV-2 variants including the highly contagious delta variant (B.1.617.2) (108) and the highly pathogenic lambda variant (C.37) (109).

**Figure 2:**
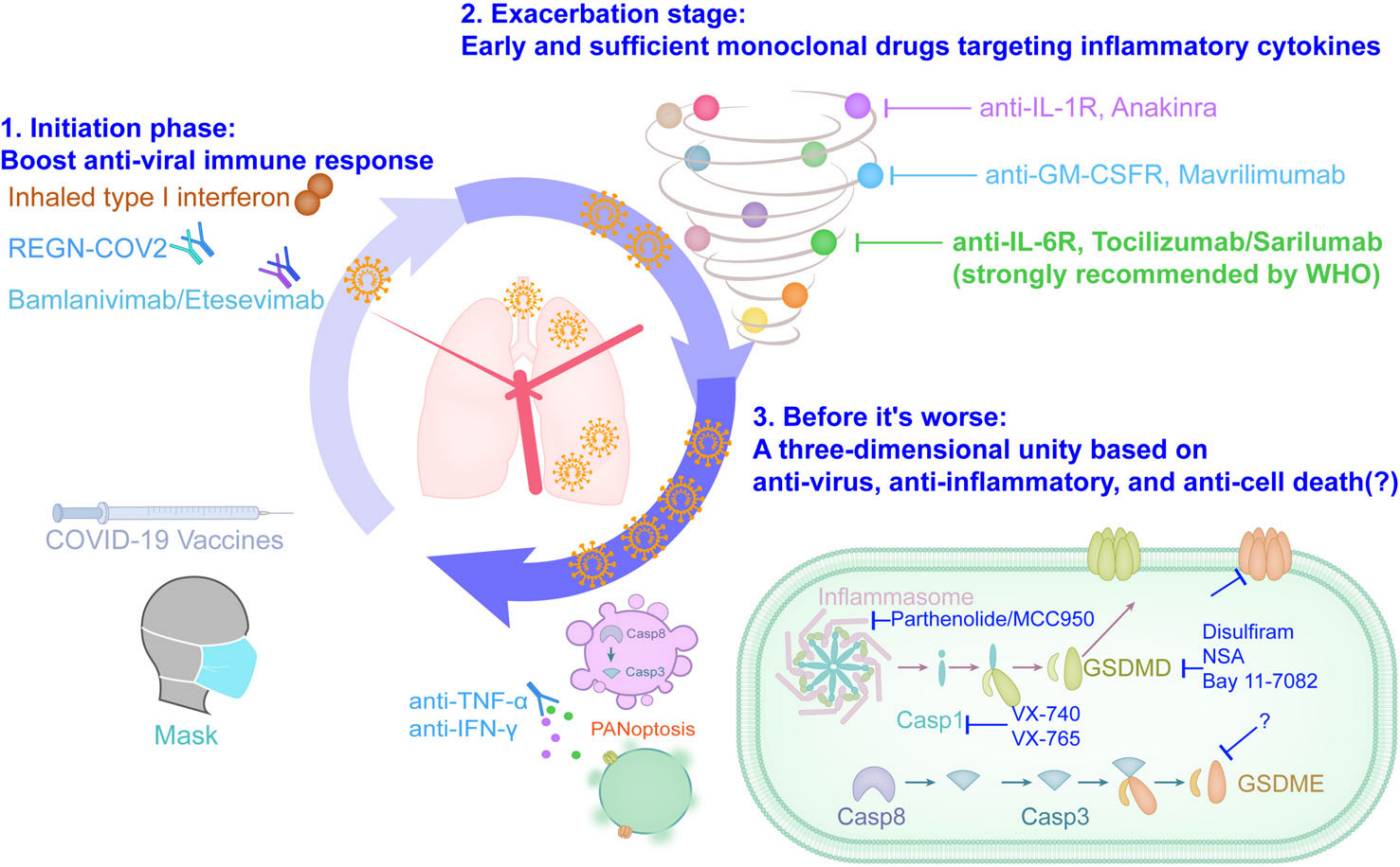
The three-dimensional integrated solution based on anti-viral, anti-inflammatory and anti-cell death slows down the ARDS clock of severe COVID-19.

From mild pneumonia symptoms to ARDS, to multiple organ failure, it is still the main cause of death in severe COVID-19 patients (14, 23, 45, 87). Reorganizing the process of inflammatory storms is not only important for understanding the progress of the disease, but also helps us to form a more complete treatment plan. Aiming at the mechanism that drives patients to progress from pneumonia to ARDS and multiple organ failure, the inflammatory storm that is gradually aggravated, we propose a new three-dimensional integrated treatment strategy for future research under ethical precursors: 1. Initiation phase: Block SARS-CoV-2 from entering cells and boost anti-viral immune response; 2. Exacerbation stage: Early and sufficient monoclonal drugs targeting inflammatory cytokines; 3. Before it’s worse: A three-dimensional unity based on anti-virus, anti-inflammatory, and anti-cell death (**Figure 2**). The drafting of this strategy is inspired by the results of published clinical trials. The single neutralizing antibody of anti-SARS-CoV-2 is not ideal (110), and the combination as a cocktail is recommended for mild patients with COVID-19 that plays a good role in blocking the infection of host cells in the early stage of viral infection (100). Tocilizumab, which is strongly recommended by the WHO, also requires a combination of antiviral agents because tocilizumab alone cannot be more effective than the placebo group (COVACTA, NCT04320615) (30), and adding tocilizumab on the basis of standard care benefit severe and critically ill patients with COVID-19 (25, 26, 107). This three-dimensional integrated treatment strategy not only highlights the combination of different drugs such as antiviral and anti-inflammatory, but also highlights the need for targeted addition of drugs at different stages of COVID-19, and calls for adding drugs to alleviate cell death before COVID-19 becomes life-threatening. At present, most of the screening inhibitors of targeted cell death are still in the cutting-edge basic research. At present, most inhibitors that target cell death is still in the cutting-edge basic research. Disulfiram, as a drug that has been approved for the treatment of alcohol addiction, has recently been reported to target gasdermin D to prevent it from making holes in the cell membrane, which can effectively alleviate the death of a mouse model of sepsis (111). Given that COVID-19 can produce an inflammatory syndrome that is similar to sepsis, whether disulfiram can be used to treat severe COVID-19 patients should be considered, and it can inhibit the Lpro protease of SARS-CoV-2P, which has the potential to inhibit virus replication (112). In addition, the combined treatment of neutralizing antibodies against TNF-α and IFN-γ in mice infected with SARS-CoV-2 can alleviate PANoptosis and protect mice from death (84). Although the existing evidence only comes from mouse models, anti-cell death is a potentially promising therapeutic idea in life-threatening infectious diseases caused by inflammatory storms including COVID-19.

The three-dimensional integrated treatment strategy including anti-viral, anti-inflammatory and anti-cell death is an ideal combination of saving the lives of COVID-19 patients, and its effectiveness needs to be repeatedly tested under the precursors of ethical and clinical research guidelines. Clinically, the definition of mild, severe and critical COVID-19 mainly refers to the imaging characteristics of pneumonia and blood oxygen related indexes, such as blood oxygen saturation and arterial oxygen partial pressure (13, 14). However, the clinical manifestations of the patient are delayed relative to the body damage. The blood biochemical test report of the patient showed a significant decrease in lymphocyte count, a significant increase in inflammation indicators (IL-6, C-reactive protein, ferritin, etc.) and an increase in blood coagulation function indicators (D-dimer, procalcitonin, thrombin time, etc.), which are potential early warning indicators for severe and critically ill patients with COVID-19 (14, 87). In addition, attempts to propose faster and more accurate COVID-19 prediction models from the aspects of clinical symptoms (113), transcriptome (114), serum protein (115), and metabolome (116) based on artificial intelligence algorithms have also been established, but they still need to be further confirmed in the future. And the limitations of these parameters and data models that are indicative of the progression of COVID-19 may also be discovered in the future exploration of the three-dimensional schedule, so that they can be further improved in a targeted manner, so as to indicate the medication window more timely and accurately.

## Author Contributions

All authors contributed to the article and approved the submitted version. YZ conceptualized and drafted the manuscript. XX drafted figures. HW edited/reviewed the manuscript.

## Funding

This work was supported by China National Center for Biotechnology Development (2020YFC0843800 to HW), Jack Ma Foundation (ZB9100000001 to HW) and the project funded by USTC Research Funds of the Double First-Class Initiative (no. YD9100002008 to YZ).

## Conflict of Interest

The authors declare that the research was conducted in the absence of any commercial or financial relationships that could be construed as a potential conflict of interest.

## Publisher’s Note

All claims expressed in this article are solely those of the authors and do not necessarily represent those of their affiliated organizations, or those of the publisher, the editors and the reviewers. Any product that may be evaluated in this article, or claim that may be made by its manufacturer, is not guaranteed or endorsed by the publisher.
